# Clinical Features, Etiopathogenesis, and Therapeutic Approaches of Acute Gingival Lesions: A Narrative Review

**DOI:** 10.7759/cureus.67129

**Published:** 2024-08-18

**Authors:** Shweta Bhagat, Priyanka Jaiswal, Sakshi V Kotecha

**Affiliations:** 1 Department of Periodontics and Implantology, Sharad Pawar Dental College, Datta Meghe Institute of Higher Education and Research, Wardha, IND

**Keywords:** acute aphthous stomatitis, acute herpetic gingival stomatitis, acute candidiasis, acute streptococcal gingivitis, acute pericoronitis, acute gingival abscess, acute necrotizing gingival lesions, gingivitis, acute gingival lesions

## Abstract

A bacterial infection is typically the source of gingivitis, a non-destructive condition that produces gum inflammation. Unlike chronic lesions, which have well-defined features and a gradual onset, acute lesions are unpleasant. Usually, the first symptoms are bleeding, swollen, and red gums. If gingivitis is not treated, it can lead to periodontitis, a more serious condition where the gums separate from the teeth. It seems that gingival disease-induced inflammation is a necessary condition for the breakdown of connective tissue attachment apical to the cementoenamel junction (CEJ). This may result in damage to the bone and soft tissue that support the teeth, leading to loosening and unstable teeth, and if the infection worsens, eventual tooth loss may occur. While one of the most prevalent inflammatory diseases in humans is plaque-induced gingivitis, there are several less common but frequently very significant non-plaque-induced gingival illnesses. Maintaining regular dental hygiene can help prevent gingivitis and make it reversible. Non-plaque-induced gingival lesions might be pathologic alterations restricted to gingival tissues, but they are also frequent signs of systemic disorders. Diagnosing patients with these lesions and creating treatment regimens for them must be the aim of the therapy.

## Introduction and background

Infection is a typical natural phenomenon that occurs when a parasite lodges and multiplies in or on host tissue. Unlike chronic lesions, acute lesions are unpleasant and have well-defined features. They also occur suddenly and last a short while. A range of processes can lead to the complicated and unique pathological entities that make up gingival disease [1]. Acute gingival lesions encompass a spectrum of conditions characterized by an abrupt onset and gingival tissue inflammation. On account of their diverse etiology, clinical presentation, and possible influence on oral health, these lesions pose an inevitable challenge in dental treatment. Surrounding the teeth and supporting tissues, the gingiva, or gums, serve as a barrier. Inflammatory signs including redness, swelling, pain, and bleeding can occur in the gingival tissues when they are subjected to irritants or damage. Numerous factors, such as trauma, infection, allergic reactions, and systemic disorders, can result in acute gingival lesions [2].

Accurate diagnosis and successful treatment of acute gingival lesions depend on an understanding of the underlying etiology of these lesions. In order to identify the precise origin of the lesion, clinicians must perform a complete assessment that may include a thorough patient history, a clinical examination, and diagnostic tests to promote optimal healing, prevent complications, and minimize the risk of recurrence [3]. Management may include conservative measures such as oral hygiene instructions, topical medications, and professional cleaning or more aggressive interventions such as systemic antibiotics or surgical intervention in severe cases. Patient education on preventive measures and follow-up care is integral to long-term oral health outcomes [4].

This review aims to provide an overview of acute gingival lesions, including their etiology, clinical presentation, diagnostic considerations, and management strategies by identifying, diagnosing, and treating acute gingival lesions more effectively by understanding the fundamental characteristics of these conditions.

## Review

Acute necrotizing ulcerative gingivitis

Acute necrotizing ulcerative gingivitis (ANUG) is a rare, non-contagious microbial infection of gingiva associated with impaired host response. Onset is sudden and affects less than 1% of the population [5]. The manifestations are interdental papillae ulceration and painful bleeding gums causing the destruction of the supporting structures. The disease is distinguished by the degeneration and sloughing of gingival tissue, accompanied by characteristic signs and symptoms [6]. Some of the terminologies are Vincent’s disease, Trench-mouth disease, necrotizing gingivo-stomatitis, fusospirochaetal stomatitis, ulcerative membranous gingivitis, acute ulcerative gingivitis, necrotizing ulcerative gingivitis (NUG) and ANUG [7].

In the fourth Century, ANUG was recognized by Xenophon who stated that Greek soldiers were characterized by sore, ulcerated, and foul-smelling mouths [8]. Necrotizing gingivitis was shown to be highly prevalent (14%) in a variety of demographics, including military units, during World War II, and moderately prevalent (2.2%) in North American populations in the 1950s [9]. It is evident that the incidence declined after World War II in industrialized nations. The clinical distinctions between chronic periodontitis, scurvy, and NUG were initially made by John Hunter in 1778 [10]. Plaut (1894) and Vincent (1896) characterized the illness and suggested that spirochetes and fusiform bacilli were its cause [11]. In the 19th century, there was an outbreak of NUG in the French army. Many "epidemics" struck the Allied troops during World Wars I and II. Necrotizing gingivitis patients, however, are often vulnerable to recurrence of the disease in the future. This is mostly because managing predisposing variables and maintaining adequate supragingival biofilm control can be challenging. The presence of NUG in epidemic-like outbreaks does not always indicate that the condition is communicable. This episode is severe. It becomes less severe, reaching a subacute stage with softer clinical manifestations. These individuals typically have a history of remissions and exacerbations [6].

Clinical Signs and Symptoms

The pathognomonic characteristics are punched-out, crater-like depressions that extend to the border of the gingiva at the crest of the interdental papillae [12]. It commences as a localized disease that becomes a generalized disease and spreads laterally from the papilla to the gingival margin, affecting both the buccal and lingual sites. A distinct linear erythema sets apart the grey pseudomembranous slough covering the crater surface from the surrounding gingival mucosa. Bleeding occurs spontaneously from the gingiva or becomes noticeable at the slightest stimulus with increased salivary production and fetid odor. It is exceptionally touch-sensitive constantly radiating and causing gnawing pain that is enhanced by chewing and consuming hot or spicy meals producing pasty-type saliva with metallic bad taste. There is local lymphadenopathy and possibly an increase in body temperature during the mild to severe stages of the illness. High temperature, rapid heartbeat, leukocytosis, anorexia, and generalized lassitude are signs of severe cases [6]. Gangrenous stomatitis and noma are the result of severe sequelae. If left untreated, NUG can proceed to necrotizing ulcerative periodontitis (NUP), which causes gingival recession and periodontium to deteriorate gradually [13].

Etiology

Role of microorganisms: Infectious pathogens are the cause of necrotizing gingival disorders. Plaut (1894) and Vincent (1896) had already established the bacterial etiology of this illness through microscopic analysis of plaque samples taken from afflicted individuals, which amply indicated the presence of fusiform bacteria and spirochetes even within the tissues. The fusiform bacteria and spirochetes are prevalent in necrotic lesions and have the tendency to penetrate connective tissue and epithelium. They can also emit endotoxins, which can destroy periodontal tissue by altering or triggering the host response. Listgarten (1965) identified four zones: the bacterial zone, the neutrophil-rich zone, the necrotic zone, and the zone of spirochetal infiltration. These zones may or may not coexist in each situation (Table 1) [14].

**Table 1:** Zones in ulcerated lesions ANUG: acute necrotizing ulcerative gingivitis

S.no.	Zones of infection within ANUG lesions (superficial to deep)
1.	The bacterial zone consists of a surface fibrous mesh consisting of leukocytes, cellular remnants, degenerating epithelial cells, and a diverse range of bacteria, such as spirochetes, fusiform, and rods
2.	Neutrophil-rich zone, which is located in between the host cells and is comprised of many leukocytes, particularly neutrophils, as well as numerous spirochetes of varied sizes and other bacterial morphotypes
3.	The necrotic zone is composed of disintegrated cells, in addition to fusiform bacteria and spirochetes
4.	Spirochetal infiltration zone, in which large- and medium-sized spirochetes penetrate the tissue while other tissue components retain their integrity and are preserved

The fact that fusiform bacilli and spirochetal organisms are consistently present in the illness, along with other species, indicates the presence of a fusospirochetal complex. This complex comprises Treponema microdentium, vibrios, fusiform bacilli, intermediate spirochetes, filamentous organisms, and multiple Borrelia species.

Role of host response: Owing to the host immune response's downregulation, all of the predisposing factors for NUG are linked to immunosuppression. It occurs due to immunosuppression alterations in the immune system and leukocyte function, markedly reduced polymorphonuclear leukocyte (PMNL) chemotaxis and phagocytosis, decreased peripheral blood lymphocyte proliferation, and elevated blood steroids [4,15].

Predisposing factors: There is insufficient data to determine the prevalence of necrotizing periodontal diseases in populations that are systemically healthy [16]. Although more than one condition is typically required to initiate the disease, predominant predisposing factors for necrotizing gingival disease are those that modify the host immune response (HIV infection). These include dietary problems, psychological stress, chronic inadequate sleep, systemic conditions (cancer, syphilis, and severe GIT disturbances such as ulcerative colitis and blood dyscrasias like leukemia and AIDS), inadequate maintenance of oral health, a previous history of necrotizing periodontal disease and gingivitis, young age, and ethnicity [2].

HIV-positive patients: Necrotizing periodontal diseases are prevalent and advance more rapidly in HIV patients. There is evidence to show that people living with HIV exhibit a greater propensity for recurrence and reduced responsiveness to mechanical and pharmaceutical periodontal therapy [17]. Necrotizing gingivitis and necrotizing periodontitis have been linked to decreased counts of peripheral CD4 lymphocytes in HIV patients [18]. As a result, the diagnosis of necrotizing periodontal disease should raise the possibility that the patient may be HIV positive, and the affected subjects should undergo HIV screening [16].

Malnutrition: Necrotizing periodontal diseases have also been linked to malnutrition, particularly in underdeveloped nations. "Protein-energy malnutrition" has been proposed as the basis for this relationship, suggesting a significant decrease in important antioxidant nutrients and a modified acute-phase response to infection. Additional effects include histaminemia, elevated free cortisol in blood and saliva, abnormalities in mucosal integrity, and an inverse relationship between helper and suppressor T-lymphocytes [19].

Psychological stress and insufficient sleep: Necrotizing gingivitis has been linked to severe psychological stress and acute stress circumstances [2]. Necrotizing periodontal disease can be more common in certain circumstances. These include wartime military people, recently enlisted military personnel, drug abusers having abstinence syndrome, students during test periods, and patients suffering from depression or other psychiatric disorders [20]. Not only is the immune response changed during these stressful times, but the subject's behavior is likewise, which may result in poor diet, poor oral hygiene, increased tobacco use, decreases in salivary flow, and gingival microcirculation as well as elevations in 17-hydroxycorticosteroid levels in the serum and urine, which are linked to changes in polymorphonuclear leukocyte, lymphocyte function, and marked increase in periodontal bacteria like *Prevotella intermedia *[21]. Significantly higher degrees of agitation, anxiety, irritability, depression, or emotional dysregulation have been linked to elevated urine levels of 17-hydroxycorticosteroid compared to healthy or treated patients [16]. Polymorphonuclear leukocytes in patients with necrotizing gingivitis also displayed changed functions due to decreased phagocytic, chemotactic, and bactericidal capacities [15].

Inadequate oral hygiene: Plaque buildup has been linked to necrotizing periodontal disease, but it can also result from ulcers and crater lesions, which can make brushing difficult due to pain [2].

Pre-existing gingivitis: It always develops on top of pre-existing periodontal pockets and chronic gingival disease. Vulnerable locations such as deep periodontal pockets and pericoronal flaps provide an environment that is conducive to the growth of anaerobic fusiform bacilli and spirochetes [6]. Necrotizing periodontal disease typically develops on top of an underlying periodontal condition, most commonly chronic gingivitis. NUG frequently occurs in gingival tissue that has been traumatized by opposing teeth in malocclusion.

Alcohol and tobacco consumption: A 98% of patients having NUG were smokers. The risk factor for necrotizing periodontal disease is smoking, and the majority of HIV-negative patients who were diagnosed with the condition were smokers [22]. Smoking's effects on inflammation and tissue response are attributed to the inhibition of polymorphonuclear leukocyte and lymphocyte function as well as the vasoconstriction of gingival blood vessels caused by nicotine. Necrotizing periodontal disease promoting physiological and psychological variables has also been linked to alcohol drinking [2].

Young age and ethnicity: Age is typically associated with other risk factors, such as stress and smoking [2]. Necrotizing periodontal disease strikes even younger persons in developing nations, and the most common risk factors are infections and malnourishment [19].

Diagnosis 

Necrotizing gingivitis is mostly diagnosed based on clinical signs, such as the development of ulcers and necrosis in the free gingiva. These lesions show a characteristic "punched-out" look and typically begin near the interdental papilla. Furthermore, there may be a marginal erythema that distinguishes between healthy and diseased gingiva. This condition is known as "linear erythema." The marginal gingiva may become affected by these necrotic lesions. The anterior teeth, particularly those in the jaw, are the most common site. Gingival bleeding is a common observation in necrotizing gingivitis and typically occurs spontaneously or with very little stimulus. Depending on the size and severity of the lesions, pain typically starts quickly and might vary in intensity [3]. A pseudomembrane over the necrotic area is seen. Fever along with discomfort are the common symptoms in the patients [23].

*Management of Necrotizing Periodontal Diseases* 

Therapy aims to halt the disease process and tissue loss, with management of the patient's general pain and discomfort, which prevents them from eating and from practicing appropriate oral hygiene. To get rid of the soft, mineralized deposits, a thorough superficial debridement should be the first step.

First visit: The distribution, defining feature, and potential involvement of the oropharyngeal region of NUG are assessed in the oral cavity. Periodontal pockets, local variables, and the existence of pericoronal flaps are all taken into consideration while evaluating oral hygiene. The care given in the first appointments consists of using a moistened cotton pellet on affected areas. The pseudomembrane and nonattached surface detritus are removed following the application of topical anesthetics. Ultrasonic scalers are used to eliminate supragingival calculus after the area has been cleaned with warm water. At this point, subgingival scaling and curettage are not advised to reduce the risk of escalating the acute symptoms. Procedures like teeth extractions and periodontal surgeries are delayed for at least four weeks till the patient is without symptoms. Patients who exhibit systemic signs or symptoms, local lymphadenopathy, or moderate to severe NUG are prescribed 500 mg of amoxicillin orally every six hours for 10 days. Metronidazole (500 mg twice daily for seven days) or erythromycin (500 mg every six hours) are other substitutes. It appears that the first-choice treatment for NPD is metronidazole, which should be taken three times a day due to its effectiveness against spirochetes [21].

Since intralesional bacteria are common and topical administration does not provide an adequate intralesional concentration of antibiotics, topical application of antibiotics is not recommended. Other oxygen-releasing drugs, such as hydrogen peroxide, have a long history of use in the treatment as well. Equal parts of 3% hydrogen peroxide and warm water were used as a mouth rinse and for debridement in necrotic areas. The benefits of hydrogen peroxide may stem from mechanical cleaning as well as the oxygen release’s impact on anaerobic bacterial flora. When teeth brushing is difficult to perform, rinsing twice a day with a 0.2% chlorhexidine solution is an extremely efficient method for reducing plaque buildup. It also helps with self-maintained dental hygiene in the initial weeks of therapy. Symptoms are reduced in a matter of days with the right care within five days.

Second visit: As the symptoms lessen after the first visit within one or two days, systematic subgingival scaling should be maintained. After ulcers have been healed, restoration margins should be corrected and finishing of restorations and root surfaces should be done. Oral hygiene and patient motivation are added to local treatment. Treatment for minor craters may involve gingivectomy, along with gingivoplasty. Periodontal flap surgery or even regenerative surgery are better possibilities to treat extensive craters [6].

Maintenance phase: Necrosis and acute symptoms vanish upon completion of the acute phase treatment, i.e., approximately five days after the second visit. Although some flaws typically continue, the gingival craters shrink and the previously necrotic areas recover. Plaque control techniques are instructed to the patient. Patient counseling is done on diet, smoking, and other habits that may cause recurrence.

Healing: The typical NUG lesion responds to treatment by changing during the healing process. When the pseudomembrane is removed, the gingiva's underlying red hemorrhagic crater-like depression is revealed. The following stage is characterized by a decrease in the bulk and redness of the crater borders and restoration of normal gingival color and contour. Finally, restoration of the typical gingival surface texture, contour, and consistency can be seen. Healthy gingiva may cover the root exposed by the acute illness [6].

Acute primary herpetic gingivostomatitis 

Acute herpetic gingivostomatitis is a condition where there is a primary infection of the oral cavity with the herpes simplex virus (HSV-1). This infection is frequently accompanied by systemic signs and symptoms. Infants and children under the age of six are susceptible to type 1 HSV-1. An asymptomatic primary infection exists. The virus enters the body through the autonomic or sensory nerves and remains latent in the neural ganglia that innervate the location. Herpes labialis, herpes genitalis, ocular herpes, herpetic encephalitis, and other secondary symptoms might arise after exposure to sunlight, fever, trauma, stress, or oral surgical operations.

Communicability

This infection is communicable. Most individuals acquire immunity as a result of a subclinical virus they contracted as children. Research has shown that HSV is present in periodontal pockets [24]. Herpes labialis is one example of a secondary herpetic skin infection that might return [25].

Clinical Features

A variable degree of edema and gingival bleeding is present in the intraoral widespread. Diffuse erythematous, shiny involvement of the gingiva, and adjacent oral mucosa can be seen. Discrete, round, grey vesicles are visible on the tongue, pharynx, gingiva, buccal, labial, and sublingual mucosae. After a day, the vesicles burst and turn into tiny, excruciating sores with a red, raised, halo-like margin with a depressed, yellowish-white, or greyish middle section. A widespread acute marginal gingivitis is a crucial diagnostic criterion for this illness. The illness takes seven to 10 days to progress. Around healed areas, there is no scarring, although there is discomfort and trouble eating and drinking. The intraoral illness may be accompanied by lip and face involvement (herpes labialis and cold sores), with vesicles and surface scale formation. Patients typically experience a high temperature along with generalized malaise and cervical adenitis symptoms [26].

Differential Diagnosis

This includes herpangina, hand-foot-and-mouth disease, aphthous stomatitis, Bechet syndrome, Pemphigus vulgaris, and Stevens-Johnson syndrome.

Diagnosis

Early diagnostic criteria for HSV infection are typically clinical. The most commonly performed diagnostic procedures are cytological smears and tissue biopsies. These are followed by detection using various techniques such as virus culture, microscopy, and imaging. Immunologic testing, which includes monoclonal antibodies or DNA hybridization techniques, is used to detect viral glycoproteins, viral genetic material, and viral antibodies. These methods are crucial for detecting HSV in lesions.

Treatment

Consists of antiviral medications right away after an early diagnosis. Palliative care involves the removal of supragingival calculus, plaque, and food residue. Antipyretic medication is used to control fever. Acyclovir suspension (400 mg orally three times per day or 200 mg five times per day within three days of onset) lessens fever, discomfort, lesions, and virus-shedding days [27]. Acyclovir slows DNA replication in HSV-infected cells but has no effect on normal cells. Antivirals, such as Famicyclovir and Valacyclovir, are also effective for three days. Systemic and local antibiotics are prescribed to stop opportunistic infections, particularly in patients with weakened immune systems. Extensive periodontal therapy must be delayed. The patient needs to be made aware of the disease's contagiousness and the need for safety measures. One can apply acyclovir cream (five times daily for five days) and mucosal ointment for short-term alleviation. Mouthwashes containing topical anesthetics, like lidocaine hydrochloride, can be used (Table 2) [28].

**Table 2:** Differential diagnosis of ANUG ANUG: acute necrotizing ulcerative gingivitis; HSV: herpes simplex virus

ANUG	Primary herpetic gingivostomatitis	Desquamative gingivitis	Chronic destructive periodontal disease	Diphtheria
Fusospirochetal complex	HSV	Few bacterial forms	Variable bacterial smears	Corynebacterium diphtheria
Punched-out necrosed papilla and marginal gingiva	Gingiva (diffused) buccal mucosa and lips	Gingiva and mucosa	Marginal gingiva	Marginal gingiva (rarely)
Pseudomembranous epithelium	Diffuse erythema and vesicular eruption	Patchy desquamation	No desquamation, but purulent material may appear from pockets	Membrane removal difficult
Adults and adolescents	Children	Adults and Women	Adults	

Acute pericoronitis 

Inflammation of the gingiva in connection to the crown of a partially erupted tooth is known as pericoronitis. It mainly affects the area of the mandibular third molar. One can have acute, subacute, or chronic pericoronitis. It is an inflammation of the surrounding gingiva and tissues that surrounds the crown of a partially erupted tooth. An inflammatory state affects the gingiva and other supporting tissues surrounding the crown of a tooth that has partially or fully erupted, particularly a distal tooth in the arch [29]. Pericoronitis can be classified as acute or chronic. Heat, discomfort, redness, swelling, and loss of function are the immediate and severe manifestations of inflammation that accompany acute pericoronitis. The symptoms of chronic pericoronitis are typically moderate and prolonged, with possible subclinical inflammation [30].

Operculum 

A soft tissue that covers a partially erupted tooth. A potential deep and large space occurs between the soft tissue and the crown. These pockets are colonized by a wide and varied bacterium and gingival inflammation nearly invariably results.

*Etiology* 

Partial eruption, oral microbiota, malposition, immediate proximity of ramus of mandible, food impaction, and poor oral hygiene are important etiological factors. Opposite teeth pressing against the operculum and having poor oral hygiene are other etiological factors. Local factors include the third molar that favors deep distal or distobuccal pocket formation. More often, these impacted molars have vertical and distoangular impactions [31]. Systemic factors include upper respiratory tract infections, tonsillitis, smoking, emotional stress, and general debilitating disease [29].

*Clinical Features* 

Distinguished by pericoronal flap inflammation and surrounding structures, the mass of the flap is increased by cellular exudate and inflammatory fluid, which might traumatize the area in case it comes in touch with the opposing jaw and prevent the jaws from closing completely. The lesion is noticeably red, swollen, and suppurating. It is painful, and discomfort radiates to the floor of the mouth, throat, and ears. The throbbing pain gets intense and is exacerbated by chewing. Halitosis, bad taste, and difficulty in closing the mouth are other features. Lymphadenitis and cheek swelling in the jaw's angle are frequent. Fever, malaise, elevated pulse, respiratory rate, and leukocytosis are some other systemic complications. When palpated, the submandibular lymph nodes could be swollen and painful [6].

Differential Diagnosis 

The clinical diagnosis of pericoronitis is based on soft tissue inflammation and the presence of infection [32]. Histological studies or radiographic examinations can be used to distinguish one illness from another. Foreign body impaction, pyogenic granuloma, peripheral ossifying fibroma, dental caries, periodontitis, periapical abscess, or granuloma are the differential diagnoses [33].

*Diagnosis* 

Involves clinical and radiographic examinations with meticulous recording of patient’s past history of disease.

Treatment 

Conservative treatment: It is used for well-localized pericoronitis affecting a tooth. It consists of using mild curettage and external pressure to debride and empty the pocket. Sterile saline solution should be used to irrigate the pocket both during and after curettage. An antibacterial solution, such as 10% povidone-iodine, can also be used for irrigation. When an abscess fluctuates, an incision should be made to drain the fluid. Until the symptoms go away, patients are instructed to clean the area frequently with warm water or saline solution. The necessity of corrective surgery should be taken into consideration when symptoms and indicators have returned. Depending on the position of the tooth in the jaw and the interactions between soft tissues, resection of some or all the pericoronal tissue (operculectomy) lowers the risk of recurrent infection. The mainstay of pain therapy should be non-steroidal anti-inflammatory drugs (NSAIDs) and oral analgesics [34].

Operculectomy: It is a surgical removal of operculum. It can be carried out with a laser, electrosurgery, or hand devices [35]. To help lessen discomfort and speed up healing from pericoronitis, an opposing supra-erupted third molar needs to be extracted soon. If, following careful inspection and evaluation, a tooth is determined to have a greater possibility for persistent infection and is not expected to erupt into a functional occlusion, extraction of the tooth or the opposing tooth may be an option. If the abscess is not visible and the pericoronitis is localized, extraction can be done immediately or can wait until the acute inflammation has resolved. If pus is present, the acute phase should be resolved before an elective extraction. In cases when mechanical damage from an opposing tooth is aggravating pericoronitis, extraction of the opposing tooth may be a viable treatment option.

Perioperative systemic antibacterial therapy is administered if an immediate extraction is required and applied to most cases of severe pericoronitis with signs of regional spread. Close observation of the results is required. Delaying extraction until the infection has fully cleared is advisable [36].

Antimicrobial regimen: The mainstay of pain therapy should be NSAIDs and oral analgesics [34]. In cases of pericoronitis where there is suspicion of systemic infection spread, antibiotics such as amoxicillin (250-500 mg three times daily for five days) and metronidazole (200-400 mg three times daily for five days) are recommended. For patients who are allergic to penicillin, alternative drugs such as erythromycin 250-500 mg, ampicillin 250-500 mg, and doxycycline 200 mg once a day for the first day followed by 100 mg every 12 hours per day are recommended.

*Sequelae* 

A pericoronal abscess can be the only sign of the involvement. There can be involvement of the retropharyngeal, posterior cervical, and submaxillary lymph nodes, depending on the degree and severity of the infection. Necrotizing gingivitis can develop from untreated pericoronitis. Serious life-threatening complications are Ludwig’s angina, cellulitis, acute meningitis, and cavernous sinus thrombosis [6].

Acute gingival abscesses 

Abscesses in the periodontium are odontogenic infections that can result from trauma, surgery, pericoronitis, periodontal infections, or pulp necrosis [37]. A gingival abscess is a small, acute inflammatory lesion. Only the marginal and interdental gingiva are affected by a gingival abscess, which is a painful localized swelling that is typically linked to foreign objects that have subgingivally impacted the gingiva. A periodontal abscess develops in a periodontal pocket connected to a periodontitis lesion, whereas a gingival abscess solely affects the marginal soft tissues of previously healthy locations. Both disorders can occur in gingiva that was previously healthy. “A localised, painful, rapidly expanding lesion involving the marginal gingiva or interdental papilla, sometimes in an area previously free of disease" is the definition of a gingival abscess by the International Workshop on a Classification of Conditions and Diseases of the Periodontal System (1999).

*Etiology* 

Conditions can develop from trauma, microbial plaque infection, or impaction of a foreign object.

*Clinical Features* 

Deep swelling of the papilla and marginal gingiva. A crimson, glossy, and smooth surface that could hurt and feel sensitive when touched. There is increased depth upon probing and the potential presence of purulent exudate. Mobility of the tooth is observed. Teeth are typically vital with no history of periodontal disease. It becomes variable within 24 hours and may have a pointed surface orifice. Spontaneous ruptures can be seen [6].

Treatment 

The goal of treating a gingival abscess is to eliminate the underlying cause and reverse the acute phase. Local or topical anesthesia is applied through infiltration. Scaling is finished whenever it is feasible to create drainage and get rid of microbiological deposits. In severe situations, a #15 scalpel blade is used to sever the fluctuant region, and light digital pressure can be used to release any effusion. Any extraneous material is eliminated. The region is lightly compressed and irrigated with warm, salted water before being wrapped in moist gauze. The patient is instructed to rinse with warm saline water every two hours after the bleeding has stopped. The area is reevaluated after a day, and if the resolution is enough, any unfinished scaling is started [6].

Acute streptococcal gingivitis 

Acute gingivostomatitis, or acute inflammation of the oral mucosa brought on by a Streptococcal infection, was initially reported by Mead in 1932. Infections with Streptococci are uncommon. Specific bacterial infections of the gingiva may be caused by *Neisseria gonorrhoea, Treponema pallidum*, Streptococci, and other organisms [16]. Streptococcus species (S*treptococcus sangius, *beta-hemolytic Streptococcus A and B*, Streptococcus viridans, and Streptococcus pyogenes*) are increased on the surfaces of teeth and gingiva in *Streptococcal gingivostomatitis* [38].

*Clinical Features* 

Following tonsillitis or an upper respiratory infection, oral lesions are characterized by a diffuse erythema of the gingiva. Widely swollen gingiva seen which may have bright crimson color with patchy, circular, or linear superficial erosions that have a smear which is white or yellow in color. The disease is localized and infrequently affects the gingival tissues in their entirety. It is an acute condition characterized by fever, malaise, and pain coupled with diffuse, red, swollen, and intensely inflamed gingiva, increased bleeding, and unusual formation of gingival abscesses with an incubation period of two to four days. Fever, headache, chills, severe tonsillitis and pharyngitis, regional lymphadenitis, and pain and sore throat are observed [17].

Differential Diagnosis

Herpetic gingivostomatitis and NUG.

*Diagnosis* 

Gram stain and Streptococci isolation could be used to confirm the diagnosis.

Treatment 

Chemotherapeutic and mechanical methods are used in anti-infective therapy to reduce or eradicate microbial biofilm or bacterial plaque. Root planing as well as subgingival and supragingival scaling are indicated. Penicillin is the recommended course of therapy for Streptococci infections. Amoxicillin should be taken at a dose of 1000 mg once daily for 10 days. Penicillin and metronidazole are the antibiotics that are advised. Treatments for Streptococcal infections have also included the use of sulfonamides and broad-spectrum antibiotics. The diagnosis, treatment, and recognition of acute gingivostomatitis are crucial steps in the management of untreated group A beta-hemolytic Streptococcal infection, which can lead to numerous severe consequences [39]. Mouthwash containing benzydamine HCL (150 mg daily) and mouthwash containing chlorhexidine gluconate (0.2%) are recommended.

Acute candidiasis 

Oral candidiasis is an infection of the oral cavity caused by *Candida albicans*. It is typically brought on by immune suppression, which can be local or systemic and include illnesses that compromise immunity, such as HIV/AIDS, chronic systemic steroid use, and antibiotic use. Candida is a normal component of the oral microflora in immunocompetent people. Approximately 30% to 60% of adults and 45% to 65% of babies have Candida species in their oral cavities. Most of these species are commensal, not pathogenic, in nature.

*Etiology* 

More than 80% of lesions have been shown to contain *Candida albicans*, the species most frequently responsible for oral candidiasis. *Candida albican*s can appear as either a yeast or a hyphal. *Candida glabrata, Candida tropicalis, Candida kruesi, Candida guillermondii, Candida lusitaniae, Candida parapsilosis, Candida pseudotropicalis*, and *Candida stellatoidea* are other species that have been linked, albeit they are far less prevalent. Malnourishment, age extremes (young children and the elderly), metabolic diseases, immunocompromising conditions, concurrent infections, radiation therapy, organ transplantation, long-term steroid treatment, antibiotic treatment, and hypofunction of the salivary glands are risk factors for the pathologic colonization of Candida [40]. Oral candidiasis is caused by Candidal species when a patient's host immunity is compromised. This disruption can occur locally and be a result of using oral corticosteroids. The fungus subsequently forms a pseudomembrane as a result of its overgrowth. In healthy patients, proper bacterial flora, and the patient's immune system prevent Candida growth. A patient's oral Candida infection frequently results in gastrointestinal involvement. Therefore, immunosuppression from recent antibiotic usage, diabetes, dentures, steroid use, malnutrition, and vitamin deficiencies frequently causes the illness. Rather than existing as a pathogenic population, the majority of these species coexist in the mouth cavity as commensals [40].

There are other forms of oral candidiasis, but the most typical and classic form is pseudomembranous candidiasis, sometimes referred to as oral thrush. White or erythematous lesions are the common oral cavity manifestation of candidiasis. Erythematous lesions include angular cheilitis, linear gingival erythema, median rhomboid glossitis, and atrophic lesions. White lesions manifest as pseudomembranous or hyperplastic lesions [40].

*Acute Pseudomembranous Candidiasis* 

Acute pseudomembranous candidiasis is the most common form of candidiasis in immunocompromised patients and neonates. Reduced salivary flow and topical steroid usage are risk factors. The condition is characterized by large areas of white patches that are easily removed with gauze, revealing an erythematous mucosal surface. Fibrin, fungal hyphae, and desquamated epithelial cells compose the pseudo membrane. The tongue, labial and buccal mucosa, gingival tissues, hard and soft palate, and oropharynx are among the areas where the lesions typically manifest without any symptoms. Patients who have symptoms include mouth burning, bleeding in the mouth, and altered taste perception.

*Hyperplastic Candidiasis* 

Usually on the buccal mucosa, hyperplastic candidiasis appears as slightly elevated, well-circumscribed white plaques that are difficult to remove. The lesions may also be nodular or speckled. The formation of the lesion appears to be associated with smoking, and quitting smoking is necessary for a full resolution. Hyperplastic candidiasis has the potential to progress to malignancy or severe dysplasia.

*Acute Atrophic Candidiasis/Erythematous Candidiasis* 

The oral mucosa, usually the palate, may exhibit generalized or localized erythema due to acute atrophic candidiasis. The erythema can be accompanied by atrophy of tongue papillae. Patients typically seek medical attention when they experience burning in their tongue or mouth. Treatment with broad-spectrum antibiotics frequently results in acute atrophic candidiasis. Other risk factors might involve corticosteroids, HIV infection, iron deficiency anemia, vitamin B12 insufficiency, and uncontrolled diabetes mellitus.

Chronic Atrophic Candidiasis

Localized erythema of the oral mucosa behind dentures is a sign of chronic atrophic candidiasis, commonly referred to as denture stomatitis. On occasion, it could be observed in connection with orthodontic appliances. Lesions are usually edematous and erythematous and restricted to the area with the denture. Prolonged usage of dentures for 24 hours a day and poor dental hygiene are the main risk factors. Angular cheilitis and chronic atrophic candidiasis are typically encountered together. Although patients may not experience any symptoms, they may report burning or soreness in their mouths [41].

*Diagnosis* 

Diagnosis is made by taking history, assessment of risk factors, and examination [42]. In addition to oral candidiasis, esophageal candidiasis is a diagnostic indicator of AIDS in many HIV-positive patients.

Management 

Topical antifungal medications (e.g., nystatin, clotrimazole, and amphotericin B oral suspension) or systemic oral azoles (fluconazole, itraconazole, or ketoconazole) can be used to treat oropharyngeal candidiasis. Non-medicated mouth rinse is prescribed. HIV-positive patients' infections typically react more slowly, and 60% of them experience another episode within six months of the first one. It is possible to try higher dosages of itraconazole (up to 600 mg/d) or fluconazole (up to 800 mg/d). Oral administration of 400 mg of posaconazole suspension twice a day has also produced good outcomes in these patients. In these patients, capsofungin 50 mg/d IV and anidulafungin 100 mg/d IV have also demonstrated exceptional efficacy. Amphotericin B has been demonstrated to be efficacious at low doses (0.3-0.7 mg/kg) [40].

Acute aphthous stomatitis 

Recurrent aphthous stomatitis (RAS) is a prevalent oral mucosal illness that causes pain. These initially appear as recurring, numerous, tiny, round, or ovoid ulcers with erythematous haloes surrounding them. They have constricted edges, yellow or grey floors, and erythematous haloes [43]. Aphthous ulcers are benign but painful oral lesions whose precise cause is unknown. Synonyms* *are aphthosis, aphthous stomatitis, benign aphthous ulcers, canker sores, RAS, recurrent aphthous ulcers (RAU), and Sutton disease.

Etiology 

There is now uncertainty regarding the etiology. The cause is thought to be idiopathic and most likely multifactorial. Immune system modifications, genetic predisposition, malnutrition, and bacterial infection have all been linked [44].

Immune dysfunction: It has been suggested that changes occur in local cell-mediated immunity. The end outcome is probably immune-mediated epithelial damage.

Many patients with COVID-19 suffer from the presence of oral ulcerative lesions such as aphthous ulcers. It has recently been reported that aphthous ulcers are an oral sign of COVID-19 infection. According to a retrospective study, 1.7% of COVID-19 patients additionally reported having intraoral pain associated with aphthous stomatitis [45].

Genetics: In certain patients, a hereditary pattern is identified.

Hematinic deficiency: Studies have shown that RAU patients are twice as likely as controls to have iron, folic acid, and vitamin B12 deficiencies. Hematinic deficiency has been identified in up to 20% of patients with RAU.

Infection: The contribution of bacteria to the development of RAU is a topic of debate. It has been suggested that pathogens like *Streptococcus sanguis and Helicobacter pylori* are involved.

Clinical Features 

The pain usually lasts three to four days. Patients may complain of a burning or itchy sensation 24 to 48 hours before ulcer development. Limited to the oral mucosa, the lesions start as prodromal burning and can last anywhere from two to 48 hours before becoming ulcerated. In this first stage, erythema appears in a small area [44]. Usually, the course consists of the development of small, painful, spherical, and well-defined mouth ulcers that heal on their own in 10 to 14 days without leaving any scars. A small white papule appears within hours, eventually ulcerates, and grows over the following 48-72 hours. The typical characteristics of individual lesions are shallow, symmetric, and spherical. The absence of tissue tags aids in differentiating RAS from illnesses that cause irregular ulcers, such as erythema multiforme (EM), pemphigus, and pemphigoid. Lesions greater than 5 mm in diameter indicate a more severe form of the disease. These lesions can persist for up to six weeks. Lesions usually arise in the weakly or nonkeratinized, loosely connected areas of the oral mucosa. The rest of the mouth should be normal. The soft palate, floor of the mouth, ventral surface of the tongue, buccal mucosa, and labial mucosa are frequently involved locations.

Diagnosis 

Clinical diagnosis is made from the clinical presentation and exclusion of other systemic disease [46].

Management 

In cases of nutritional deficiencies, replacement therapy with vitamin B12, ferritin, folate, and iron are recommended.

Topical treatment: Topical drugs may reduce pain or shorten the duration but do not prevent recurrence. Topical corticosteroid is prescribed such as 0.1% triamcinolone acetonide three to four times a day. Two other topical steroids that are suitable for use are 0.01% dexamethasone elixir and betamethasone syrup. 0.05% betamethasone dipropionate, 0.05% clobetasol propionate gel, or 0.5% fluocinonide can be administered in patients with localized ulceration. Topical anesthetics such as benzydamine hydrochloride, benzocaine, and 2% viscous lidocaine can all lessen discomfort. Orabase, a topical protective emollient base, may be administered. Topical administration of Sucralfate four times per day has demonstrated a calming effect on ulcers as it adheres. Usage of tetracycline mouthwash four times a day for five to seven days (250 mg per 30 mL) is advisable. Doxycycline gel and mouth rinses containing 0.2% minocycline gel are recommended. Steroid intralesional injections, along with the appropriate use of beclomethasone spray, are also advised [47]. 

Systemic treatment: It is recommended to swish and swallow systemic steroids such as prednisolone or betamethasone syrup. It is also possible to administer betamethasone 2-3 mg/day and prednisolone tablets 20-30 mg/day for four to eight days. It is possible to shorten the recurrence time period by administering intralesional injections every two weeks for two months after systemic therapy for 15 days. Due to side effects, long-term steroid medication should be avoided. For severe cases of RAS, IV pulse treatment at 100 mg/day for three days produces rapid recovery without the negative effects linked to long-term prednisolone use. It is advised to keep an eye on the patient during treatment.

Additional treatments that may be employed include Dapsone (an anti-infective drug) 100 mg/day, Azelastine hydrochloride 300 mg/day, Cyclosporine, Gamma globulin, Prostaglandin E-2 gel, Interferon alpha 6-9 x 106 units, Azathioprine three times/week, Etanercept 25 mg/day subcutaneously, 5 mg/kg IV, and Levamisole (150 mg/day) [47].

Laser surgery: Surgical excision of the aphthous ulcer is an additional option. Laser ablation reduces related symptoms and shortens the duration. Take Nd:YAG and CO_2 _lasers, for instance. Chemical cauterization lasers have the potential to offer prompt pain relief by disrupting nearby nerve terminals or lowering inflammatory mediators [48].

Local cauterization: Topical administration of a 0.5% hydrogen peroxide solution or a 1%-2% silver nitrate solution greatly decreases the extent of the pain [49].

## Conclusions

The review of acute gingival lesions leads to the conclusion that because of their diverse etiology and clinical presentations, these lesions pose significant challenges in clinical treatment. Acute gingival lesions can be caused by a variety of factors, such as inflammatory illnesses, trauma, infections, and allergic reactions. Accurate diagnosis, which frequently necessitates a comprehensive patient history, clinical examination, and perhaps further diagnostic testing like biopsy or microbiological analysis, is crucial to addressing acute gingival lesions. The approach of treatment varies based on the underlying cause, but in severe situations, it may involve professional debridement, topical or systemic medications, or surgical intervention. To prevent complications and encourage the best possible healing, early detection and prompt intervention are essential. Recurrence can also be significantly avoided by educating patients on good dental hygiene habits and avoiding potential triggers. To improve our understanding of the pathophysiology of acute gingival lesions and to create more potent diagnostic and treatment plans, more research is required. To address the challenges that these conditions present and enhance patient outcomes, collaboration between healthcare professionals and researchers is crucial.
